# Identitag, a relational database for SAGE tag identification and interspecies comparison of SAGE libraries

**DOI:** 10.1186/1471-2105-5-143

**Published:** 2004-10-06

**Authors:** Céline Keime, Francesca Damiola, Dominique Mouchiroud, Laurent Duret, Olivier Gandrillon

**Affiliations:** 1 Équipe Signalisation et identités cellulaires, Centre de Génétique Moléculaire et Cellulaire CNRS UMR 5534, Université Claude Bernard Lyon 1, bâtiment Gregor Mendel, 16 rue Raphaël Dubois 69622 Villeurbanne cedex France; 2 Équipe Bioinformatique et génomique évolutive, Laboratoire Biométrie et Biologie Évolutive CNRS UMR 5558, Université Claude Bernard Lyon 1, bâtiment Gregor Mendel, 16 rue Raphaël Dubois, 69622 Villeurbanne cedex France

## Abstract

**Background:**

Serial Analysis of Gene Expression (SAGE) is a method of large-scale gene expression analysis that has the potential to generate the full list of mRNAs present within a cell population at a given time and their frequency. An essential step in SAGE library analysis is the unambiguous assignment of each 14 bp tag to the transcript from which it was derived. This process, called tag-to-gene mapping, represents a step that has to be improved in the analysis of SAGE libraries. Indeed, the existing web sites providing correspondence between tags and transcripts do not concern all species for which numerous EST and cDNA have already been sequenced.

**Results:**

This is the reason why we designed and implemented a freely available tool called Identitag for tag identification that can be used in any species for which transcript sequences are available. Identitag is based on a relational database structure in order to allow rapid and easy storage and updating of data and, most importantly, in order to be able to precisely define identification parameters. This structure can be seen like three interconnected modules : the first one stores virtual tags extracted from a given list of transcript sequences, the second stores experimental tags observed in SAGE experiments, and the third allows the annotation of the transcript sequences used for virtual tag extraction. It therefore connects an observed tag to a virtual tag and to the sequence it comes from, and then to its functional annotation when available. Databases made from different species can be connected according to orthology relationship thus allowing the comparison of SAGE libraries between species. We successfully used Identitag to identify tags from our chicken SAGE libraries and for chicken to human SAGE tags interspecies comparison. Identitag sources are freely available on  web site.

**Conclusions:**

Identitag is a flexible and powerful tool for tag identification in any single species and for interspecies comparison of SAGE libraries. It opens the way to comparative transcriptomic analysis, an emerging branch of biology.

## Background

In order to characterize the molecular basis underlying self-renewal versus differentiation decision-making process we investigated the transcriptomic changes of various states related to this process, in two model systems : one derived from chicken and the other from human cells. We decided to use Serial Analysis of Gene Expression (SAGE) [1] to attain this aim, for a number of reasons including the absence of available pan-genomic DNA arrays in the chicken and the ability to compare SAGE libraries across different experiments. We therefore had to resolve two problems : tag-to-gene mapping in chicken and comparing SAGE libraries from two different species (here chicken and man).

Serial Analysis of Gene Expression is a comprehensive method for analyzing transcriptomes (i.e. the complete set of mRNAs expressed in one given biological situation at one given time point) without any *a priori *regarding the genes to be studied. It can be used with mRNAs derived from cells of any eukaryotic species. SAGE is based on the isolation of a unique sequence tag from each individual transcript and on serial concatenation of several tags into long DNA molecules. Sequencing of concatemer clones reveals individual tags and allows quantification and identification of transcripts. Tag counts are digitally archived and statistically significant comparisons of expression levels can be made between tag counts derived from different populations of cells.

An essential step in SAGE library analysis is the unambiguous assignment of each 14 bp tag to the transcript from which it was derived. This process, called tag-to-gene mapping, represents a step that has yet to be completed in the analysis of SAGE libraries. The automated version of this process mostly involves extracting "virtual tags" from sequence databanks : these virtual tags are predictions of the 14 bp sequences that might be produced by a SAGE experiment. The quality of the databanks from which the virtual tags are extracted represents a limiting step in this process. Ideally, the databanks should represent the complete collection of each and every transcript, fully sequenced and annotated. This clearly has yet to be achieved for most species, and therefore one must use the available information that comes mainly from large EST (Expressed Sequence Tags) projects.

Different resources have already been described for tag identification in human and mouse, including the SAGE Map [2], the SAGE Genie [3], the Melbourne Brain Genome Project [4], the Mouse SAGE site [5] and the Human Transcriptome Map [6] web sites, but fewer resources are available for tag-to-gene mapping in other species. Nevertheless, a very large number of species have been subjected to a SAGE analysis (for an up to date bibliography, see the SAGEnet web site [7]) and actually the SAGE Map web site hosts a SAGE tag to UniGene mapping for 11 species (*Arabidopsis thaliana*, *Bos taurus*, *Homo sapiens*, *Medicago truncatula*, *Meleagris gallopavo*, *Mus musculus*, *Pinus taeda*, *Rattus norvegicus*, *Sus scrofa*, *Triticum aestivum and Vitis vinifera*). However, this site doesn't include tag to UniGene mapping for several other species for which numerous EST and cDNA have already been sequenced. This is the reason why we designed and implemented a freely available tool for tag identification that can be used in any species for which transcript sequences are available. It can include both complete cDNAs and EST cluster sequences and allow to interrogate the database according to the source of data, to assess the quality of virtual tags derived from different transcript sequences. In this paper we describe the use of this tool for the chicken (*Gallus gallus*) where a large EST sequencing effort was completed [8].

In order to allow rapid and easy storage and updating of data and, most importantly, in order to be able to query the results using sophisticated combinations of criteria, we have designed a relational database structure. We implemented this relational database called Identitag using the freely available MySQL database management system (DBMS) [9].

One important function of Identitag is the possibility to compare the tags obtained in a given species to their counterpart present in another species. This allows a direct comparison of SAGE transcription profiles obtained from different species. To the best of our knowledge, this is the first tool that allows SAGE libraries interspecies comparison : this open the way to comparative transcriptomic analysis.

Here we describe the use of Identitag for chicken SAGE tag identification as well as for chicken to human interspecies comparison.

## Implementation

### Database for tag identification : Identitag

#### Database organization

**Figure 1:**
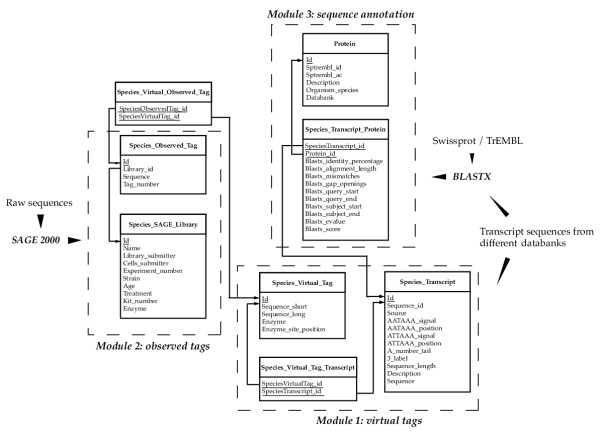
**Identitag relational schema. **This figure provides a schematic view of the Identitag tables and their relationships. Identitag can be depicted as three interconnected modules represented in this figure. For a more precise description see data dictionary (available on Identitag web site). The term "Species" could be replaced by any specific species for which transcript sequences are available. The different sources of information needed for completing Identitag are also shown. The minimum number of files consist of : one file containing tag sequences (extracting from ditag concatemers with a software like SAGE 2000), a Fasta file containing transcript sequences from the species considered and a file containing results of their comparison with protein databanks (using BLASTX).

The Identitag relational schema is presented in Figure 1, and a complete data dictionary of the database is available on the Identitag web site . We implemented this database using the freely available and cross-platform Mysql DBMS. A perl script which generates the SQL script creating Identitag tables according to the name of the species considered, is also available on Identitag web site.

#### Completing the database

Various sources of data presented in Figure 1 are needed to complete Identitag. Transcript sequences in Fasta format and a file resulting from their comparison with protein from databanks using the BLASTX algorithm [10] are needed for completing the first and third Identitag modules. For chicken Identitag we used various transcript sequences : 3425 chicken mRNA from Genbank (extracted using *query *software [11]) and 88504 SIGENAE chicken EST cluster consensus sequences (INRA, M. Douaire, P. Deshais and C. Klopp, personal communication). As consensus sequence orientation is not always known, we used both sequences and their reverse complementary. Then we could assess the correct orientation of the sequences using various sources of information stored in Identitag database (see results section). For completing the second module, a flat file with tag sequences and their relative frequency is required, for each library. So far in chicken Identitag we have stored four different libraries generated from normal chicken immature erythroid progenitors called T2ECs [12]. The first two libraries were generated from self-renewing T2EC cells and from T2EC cells induced to differentiate for 24 hours respectively [13]. The last two libraries have been generated from T2EC cells treated with two inhibitors of the MEK-1 signaling pathway which is important for maintaining self-renewal (S. Dazy et al, in preparation). For these four libraries, we used SAGE 2000 software [14] in order to extract tags from concatemer sequences : we generated 4 files for the 4 SAGE libraries considered, with 17853, 19736, 16631 and 11669 tags respectively. 6440 different tags appear more than once in these 4 libraries.

Several programs were then used for loading these data into Identitag database. They are written in Perl and Shell and are available on Identitag web site. A Shell script that allows to launch all these programs is also available on this web site : it asks for all information required by these different programs, then launches all programs with adapted arguments and loads the files generated by these programs in the corresponding Identitag tables. The creation and completion of Identitag tables with these programs was successfully tested on SUN, Linux and Mac OS X operating systems.

#### Querying the database

The completed database can be interrogated using SQL (Structured Query Language) and allows a number of tag identification procedures to be launched (see for example the procedure described in results section).

#### Redundancy reduction

When several transcripts identify a same tag, these transcripts are compared with each other using Blastclust [15] to determine whether they correspond to redundant sequences of the same transcript or to different transcripts. We consider that two sequences are redundant if they share more than 95% similarity over more than 100 bp.

### Database for interspecies comparison of SAGE libraries

**Figure 2:**
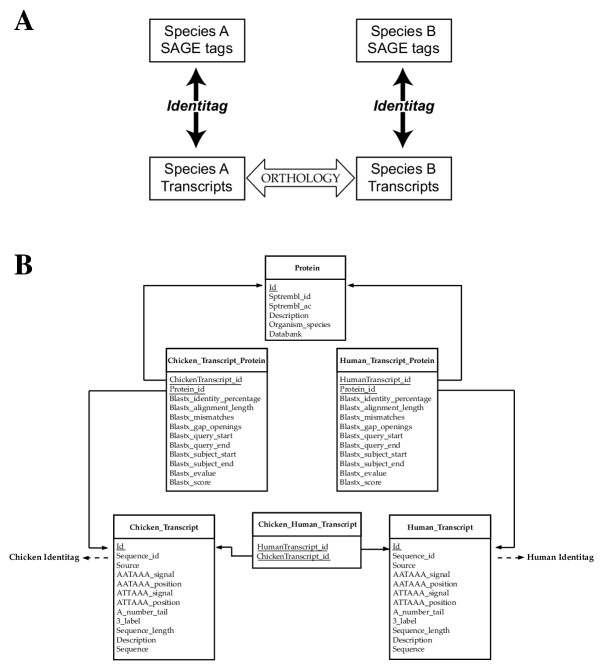
**Identitag for interspecies comparison of SAGE libraries. **A : General structure behind the process of interspecies comparison of SAGE libraries. B : Detail of the connection between two Identitag databases for generating a tool for SAGE libraries interspecies comparison (example provided for a chicken to human comparison).

We connected two Identitag databases from two different species by using orthology relationships between transcripts that identify SAGE tags (Figure 2A).

#### Design of the orthology relationship

**Figure 3:**
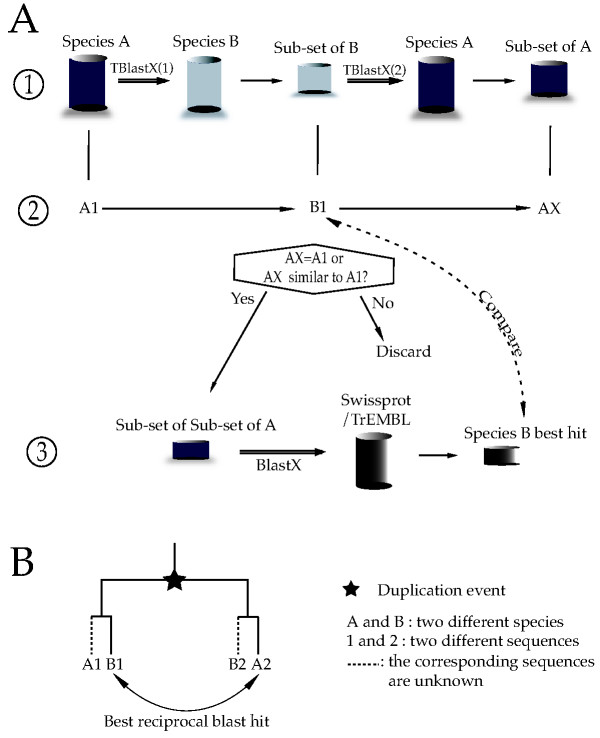
**The orthology relationship. **A. Design of the orthology relationship. Step 1 : Two reciprocal TBLASTX for comparing species A and species B transcript sequences. Step 2 : We conserve only the pairs of transcript sequences originating consistent TBLASTX(1) and TBLASTX(2) results. Step 3 : We consider previously obtained pairs in order to limit erroneous assignment of orthologous pairs for paralogous ones. B. The best reciprocal TBLASTX hits might correspond to paralogs. This figure provides an example of a phylogenetic tree where the best reciprocal TBLASTX hits correspond to paralogs because several transcript sequences are unknown (represented with dotted lines). To avoid such erroneous assignment of orthologous pairs we followed reciprocal best BLAST by another step (figure 3A, step 3) considering that even if the transcript sequences A1 and B2 are unknown, one of their corresponding proteins might be in a protein databank.

We designed a method for identifying transcript sequence pairs that are putatively orthologous between the two species considered. This method (described in figure 3A and in text below) is an approximation of the search for reciprocal best BLAST hits for two datasets with redundancy and that do not represent the entire transcriptome of the two species considered.

The first step of this method (Figure 3A, step 1) consists of two reciprocal TBLASTX. First, we compare each species A transcript with a databank containing species B transcripts, using the TBLASTX algorithm [10] (Figure 3A, TBLASTX(1)). We store all the best hits for which the corresponding E-value is less than 0.001 : all these sequences form a subset of species B transcripts. Second, we compare each sequence from this subset with a databank containing species A transcripts, using the TBLASTX algorithm (Figure 3A, TBLASTX(2)). We further consider corresponding best hits harboring an E-value lower than 0.001, they form a subset of species A transcripts that are considered for further analysis.

The second step (Figure 3A, step 2) consists in staring only pairs of transcript sequences sufficiently similar between TBLASTX(1) and TBLASTX(2). For example, transcript sequence A1 is similar to transcript sequence B1 (result provided by TBLASTX(1)), and transcript sequence B1 is similar to transcript sequence AX (result provided by TBLASTX(2)). If the two transcript datasets were complete and non-redundant, X should be equal to 1 : when that is the case, A1 is paired with B1. If not, we search if AX transcript sequence is redundant with A1, with the same criteria as described above to asses if two sequences are redundant. If AX is similar to A1, we further consider the pair A1-B1. If not, the A1-B1 pair is discarded from further analysis. We use the same method for each transcript pair obtained in first step.

If the set of transcripts from species A and B are not complete, the best reciprocal hits might correspond to paralogs (see figure 3B). To limit this risk of erroneous orthology assignment we consider the pairs stored in previous step: we compare the species A transcript sequence from each pair with species B proteins from SwissProt and TrEMBL databanks, using the BLASTX algorithm (Figure 3A, step 3). We compare the best resulting hit (a protein from species B) with the species B transcript putatively orthologous to species A transcript : if these two sequences are similar (i.e. they share more than 95% similarity over more than 100 bp), we consider the pair of the species A transcript and the species B transcript as a pair of orthologous sequences. If these two sequences are not similar, it means that protein databanks contain a species B sequence that is more similar to species A transcript than the species B sequence found using best reciprocal hit. Thus the pair of the species A transcript and the species B transcript might correspond to a pair of paralogous sequences.

These three steps allow us to obtain pairs of transcripts which are probably orthologous, by trying to eliminate erroneous assignments of orthology for paralogous sequences instead of orthologous ones. However a limiting aspect of this method is the identification of only 1-1 orthology relationships : if one transcript sequence from species A has several orthologous sequences in species B this method will only identify one of the pairs of orthologous sequences. The scripts that implement this method are available on Identitag web site.

We applied this method to chicken and human. For this we used chicken transcript sequences from Genbank, chicken SIGENAE EST cluster consensus sequences and human transcript sequences from Refseq release 2 (19902 mRNA sequences, with the accession prefix "NM_").

#### Connecting two Identitag databases by using this relationship

The database for interspecies comparisons of SAGE libraries is composed of two Identitag databases for tag identification in two different species, and connected through the SpeciesA_SpeciesB_Transcript table (see for example the organization of the database for chicken-human interspecies comparison in Figure 2B). A shell script available on Identitag web site asks for all information required. Then it searches for putative orthologous sequences using the method previously described, creates the table SpeciesA_SpeciesB_Transcript, and then loads corresponding data in this table.

## Results

### The use of Identitag for identification purpose

#### Database organization

Identitag can be depicted as three interconnected modules, as presented in Figure 1.

The first module stores data concerning transcript sequences and virtual tags extracted from these sequences. The Species_Transcript table contains transcript identification (identification number and the databank from which they originate), together with information that can then be used for assessing the quality of the "virtual" SAGE. The quality of virtual tags depends on the source of the transcript sequence from which it was derived (e.g. the sequence quality of mRNA is higher than that of EST clusters), this is why this information is stored in the Species_Transcript table. Other information can also be used for assessing the quality of the "virtual" SAGE. This information allows one to assess if a transcript sequence is complete and if its orientation correct. This includes the presence or absence of different polyA signals (AATAAA and its most common variant ATTAAA) as well as their localization along the sequence and the length of the possible poly A tail. This length corresponds to the longest poly A stretch among the 50 last base pairs of the transcript sequence : this calculation allows us to take into account the polyA tail even if there are some bases belonging to a cloning vector or sequencing errors at the end of the corresponding transcript sequence. Some of the EST we used were labeled as constructed and sequenced from the 3' region. When available this information was stored in the database. The Species_Transcript table is linked with an NN relationship to the Species_Virtual_Tag table. This table contains virtual tags extracted from the transcript sequences and information about how the tags were extracted. This includes the anchoring enzyme considered (e.g. NlaIII) and the position of its recognition site in the transcript sequence : the Species_Virtual_Tag table stores both the 10 bp sequence immediately downstream of the most 3' anchoring enzyme recognition site and 10 bp sequence downstream of the next-to-last anchoring enzyme recognition site. Indeed, the cutting enzyme may on rare occasions (0,1 %, [16]) cut not its most 3' but its recognition site that is just 5' from the last one (called next-to-last). Both conventional 10 bp tag sequences as well as virtual long-SAGE 17 bp tag sequences [17] are stored in the Species_Virtual_Tag table.

The second Identitag module allows storage and retrieval of the experimental part of the SAGE experiments. It consists of a table containing information about the construction of SAGE libraries (Species_SAGE_Library table), connected with a table that stores tag sequences from that SAGE library and their corresponding count (Species_Observed_Tag table).

The connection between modules 1 and 2 leads to a direct comparison between observed and virtual tags : it is the key to the tag identification procedure. Only perfect matches are allowed at that stage.

The third Identitag module allows annotation of transcript sequences from which virtual tags are extracted, via their similarity to known proteins. For this purpose we compare each transcript sequence with the protein sequences from Swissprot and TrEMBL databanks, using the BLASTX algorithm [10]. For this sequence comparison we only consider the same transcript sequence orientation as the orientation that we used for tag extraction. When available, the best BLASTX hits (harboring an E-value < 0.001) are stored in Identitag (in the Protein table), together with different BLAST statistics (stored in Species_Transcript_Protein table). This information can then be used for assessing the quality of the annotation.

Identitag web site provides all scripts necessary to build a such Identitag database and to load all data into this database, for any species from which transcript sequences are available. To do this one need data represented in figure 1. After running the main script one just have to answer all questions asked by the script and then all tables of the database are created and completed, for the species considered.

#### Various identification situations

**Figure 4:**
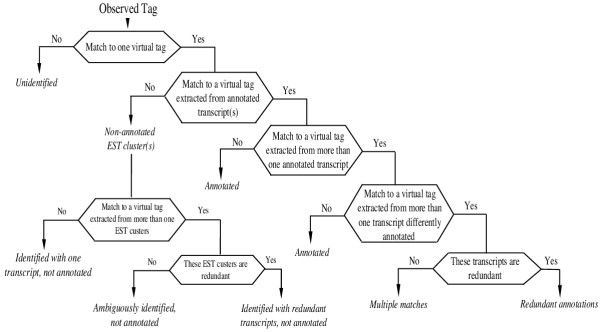
**Identitag as a tag identifier. **A : An example of identification process using Identitag. This process was used to identify SAGE tags from four chicken libraries ([13]; S. Dazy et al, in preparation).

Identitag can be interrogated in various ways, using different identification criteria concerning the quality of the virtual SAGE and the annotation provided. For example, the interrogator can use the following step-by-step procedure (Figure 4), for each observed tag from the SAGE experiment considered (an example of each identification situation is provided in table 1) :

1. If the observed tag matched to no virtual tag, then it is declared unidentified and the identification process is aborted : approximately 25% of the tags belonging to the four chicken SAGE libraries we have studied are unidentified. If this is not the case it means that the observed tag corresponds to a virtual tag extracted from one or more transcript sequence(s) ; the identification process proceeds to the next step.

2. If these transcript sequences are not annotated (i.e. they do not have any BLASTX hit with an E-value < 0.001), the observed tag is identified as matching non-annotated EST cluster(s) : 37% of the tags belonging to the four chicken SAGE libraries we studied correspond to non-annotated EST cluster(s). Then we distinguish tags identified as matching one EST cluster (57% of these tags) or more than one EST clusters. For tags corresponding to more than one EST clusters, we compare these sequences to determine whether they are redundant or not. Indeed, there could be redundancy in transcript databanks based on EST clusters due to the threshold for sequence similarity being to high to assign different EST to the same cluster. Therefore, there could be different EST clusters corresponding to the same transcript. This is the reason why we use the procedure described in implementation section to identify sequence redundancy. Among the tag identifications corresponding to more than one EST clusters, 19% correspond to redundant sequences. Finally, 65% of identifications corresponding to non-annotated EST cluster(s) are unambiguous (one corresponding EST cluster or more than one but redundant corresponding EST clusters). If the observed tag does not correspond to previous identification cases, it means that the observed tag corresponds to a virtual tag extracted from one or more annotated transcript sequence(s) and the identification process proceeds to the next step.

3. If there is only one annotated transcript sequence from which this virtual tag has been extracted, then the protein name (i.e. the description field corresponding to its Swissprot or TrEMBL accession number) is used to identify this tag and the identification process is stopped : 26% of the tags belonging to the four chicken libraries we studied correspond to this identification case. We call these tags "annotated tags". When this is not the case it means that the observed tag corresponds to a virtual tag extracted from different annotated transcript sequences ; the identification process proceeds to the next step.

4. If the different transcript sequences have the same annotation (i.e. the same best BLASTX hit), then the protein name is used as the identification and the identification process halts. This case is mainly due to transcript databank redundancy. Thus by using annotation to identify SAGE tags, we reduce the number of multiple matches. As in previous identification situation we designate the corresponding tags as "annotated tags" because their annotation is not ambiguous : 4% of the tags belonging to the four chicken libraries we studied correspond to this identification case. When this is not the case it means that the observed tag corresponds to a virtual tag extracted from several transcript sequences differently annotated ; the identification process proceeds to the next step.

5. The last case corresponds to an observed tag matching to more than one transcript sequences with more than one different annotations. This corresponds to 8% of the tags belonging to the four chicken SAGE libraries we studied. By using annotation to identify SAGE tags, we reduce the number of multiple matches that may occur because of redundancy in transcript databanks. Nevertheless, some of these multiple matches remain. This may occur because there is redundancy in protein databank, thus the redundant transcripts can be differently annotated : this leads to a multiple match. It also appear when redundant transcripts match to the same protein but in different species. Indeed the annotation is provided by a BLASTX against Swissprot and TrEMBL databanks (with all species considered) : thus we could annotate transcripts for which we don't already know the corresponding protein in the species considered, but which is identified in another species. However, this method presents the drawback of causing multiple matches when redundant transcripts match to the same protein in different species. This case is considered as a multiple match because the best BLASTX hits of transcripts identifying the same tag are different. To reduce these two cases of ambiguity, we align the different transcript sequences identifying the same tag (see implementation section) : according to the sequence similarity between them we could avoid these cases of multiple matches. Among the 521 identifications corresponding to more than one transcript sequences with more than one different annotations in our four chicken libraries, 28% could be discarded by this method. The cases of multiple matches remaining occur presumably mainly due to different transcripts that really have the same tag.

**Figure 5:**
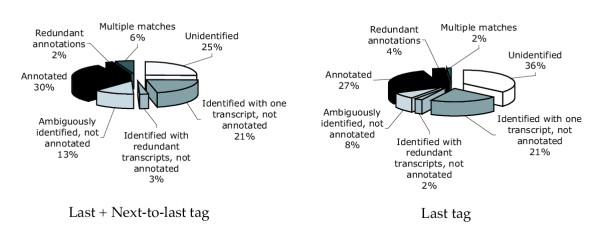
**Repartition of the different identification situations. **Repartition of the different situations exemplified in table 1 on 6440 different tags obtained in the total of four chicken SAGE libraries ([13]; S. Dazy et al., in preparation).

We provide an example illustrating the repartition of these different situations regarding tag identification in the chicken libraries we studied (figure 5). When we don't consider the next-to-last tag during the identification procedure, we reduce the number of multiple matches, but increase the number unidentified tags.

This process could be pursued further by discriminating between multiple matches, using criteria concerning the quality of the virtual SAGE, e.g. the quality of the transcript sequences (cDNA or EST), the tag position in the transcript sequence (last or next-to-last) and/or the availability of the transcript sequence end.

One can also consider a process for tag identification based only on high quality tag identifications. For example, one could obtain an identification based solely on cDNA sequences, or only using tags appearing at the last position. One can also use any logical combination of the above criteria.

### The use of Identitag for interspecies comparison of SAGE libraries

In order to directly compare SAGE libraries performed in chicken (4 libraries performed in our lab until now) with those performed in human (273 libraries available as of 08/03/04 on SAGE Genie web site : [3]), one first needs to associate each chicken tag with its human counterpart. For this we decided to connect the chicken Identitag to its human counterpart. This connection relies upon the concept of orthology (Figure 2A) [18]. Two genes in two different species are said to be orthologous if they diverged after a speciation event. It is important to note that conservation of function is not part of the definition of orthology, but rather its consequence. It can also be envisaged that after the speciation event, the function of the resulting genes diverged in the two species. A tool for interspecies comparison of expression data would be very helpful for investigating such questions, and notably the level of conservation in expression patterns of orthologous genes, a task that has only begun using DNA arrays ([19,20]).

The structure of Identitag was originally designed with this purpose in mind and therefore chicken and human Identitag could easily be connected together (Figure 2B). For example, this procedure will allow the chicken GATA-1 tag (GGGGACCCCG) to be associated with its human counterpart (GCCTCCAGAG) via the chicken transcript GATA-1 <-> human transcript GATA-1 orthology. We designed a method for identifying transcript sequence pairs that are putatively orthologous between the two species considered. This method (described in figure 3A) is an approximation of the search for reciprocal best BLAST hits for two datasets with redundancy and that do not represent the entire transcriptome of the two species considered. We first perform two reciprocal TBLASTX between species A and species B transcript sequences (TBLASTX(1) and TBLASTX(2)). We then conserve only the pairs of transcript sequences originating consistent TBLASTX(1) and TBLASTX(2) results. We finally consider previously obtained pairs in order to limit erroneous assignment of orthologous pairs for paralogous ones. For a more precise description of the design of this design of orthology relationship see implementation section.

Among the 3500 transcripts corresponding to unambiguously identified tags from our 4 chicken libraries (either annotated or unambiguously identified by EST cluster(s)), 1190 have a human orthologue as previously defined. Therefore the corresponding tags can now be translated into their human counterpart and thus SAGE libraries from two different species can be compared.

## Discussion

We have designed and implemented a tool allowing the identification of SAGE tags, based on a relational database. This structure allows to use Identitag in two different ways.

First one can precisely choose identification criteria and obtain only the tag identifications provided by using these criteria. One can specify different criteria and thus determine the quality of the identification : e.g. identification generated from EST clusters or mRNA sequences, from last or next-to-last tag, presence of the 3' end of the transcript sequence that could be inferred through the presence of a 3' label or a poly A tail and/or signal. One can also specify different criteria allowing the quality of the annotation to be controlled, e.g. quality of the similarity between transcript and protein sequences through BLAST parameters, quality of the protein sequence used to annotate the transcript via the databank from which the protein originated (Swissprot or TrEMBL) and its species (an annotation with a protein from the same species than that we consider is more accurate than with a protein from another species). All these criteria can be combined allowing the investigator to perform sophisticated interrogations of the database. To the best of our knowledge it is the first tool that allows the user to precisely adjust the identification parameters depending upon its needs.

The second way to use Identitag is to ask for all identifications available, for example for a tag of interest, and how these identifications have been generated : it is then possible to consider all these identifications and to further choose among the different identifications if necessary.

These two different ways of using Identitag can be used for any species for which transcript sequences are available. Identitag is an open source tool, the programs necessary to build and run the database are available on the Identitag web site . Identitag can therefore be used to build a tag-to-gene mapping procedure in any species, using a flat file containing transcript sequences and a BLASTX file results as input of these programs.

Identitag was successfully used for tag-to-gene mapping in chicken. It played a key role for allowing biological interpretation of the SAGE libraries obtained from normal chicken erythroid progenitor cells and allowed us to better understand the changes underlying the self-renewal versus differentiation-making process in these cells [13]. Among the identifications provided by Identitag, a few were investigated further and the vast majority of these identifications were subsequently confirmed by real-time PCR [13]. Identitag has also been successfully used for tag-to-gene mapping in Bombyx mori (J. Briolay et al, in preparation).Identitag is currently in use to identify human tags from SAGE libraries generated in order to investigate the molecular basis underlying the self-renewal versus differentiation decision-making process in human cells.

The next step will be to compare gene expression patterns between our chicken and human model systems, in order to study the possible conservation of the molecular basis of self-renewal during evolution. Comparisons of gene expression between two organisms have recently been initiated with DNA arrays ([19,20]). But it is one on the main limitations of DNA arrays that comparisons between experiments done in different laboratories (not to mention on different species) are at best approximate. It is one of the main advantage of the SAGE technique for which results can be compared without the need for sophisticated and approximative normalization procedures. The SAGE technique is therefore ideally suited for quantitative comparisons to be performed between different libraries made from different cell types in different laboratories. We therefore expect that Identitag will become a standard tool for comparative transcriptomic analysis using SAGE data, an emerging branch of biology consisting in the comparison of large scale transcriptomes obtained from various cell types belonging to different species.

## Conclusions

Identitag is a flexible and powerful tool for tag identification in any single species and for interspecies comparison of SAGE libraries. It opens the way to comparative transcriptomic analysis, an emerging branch of biology.

## Availability and requirements

• **Project name: **Identitag

• **Project home page: **

• **Operating system(s): **SUN, Linux, Mac OS X

• **Programming languages: **Perl, Bourne Shell, MySQL

• **License: **GNU GPL

## Authors' contributions

CK participated to the design of Identitag, implemented Identitag, and participated to the biological validation of Identitag with SAGE libraries. FD constructed the first two SAGE libraries with which Identitag was tested and participated to the biological validation of Identitag with these data. LD and DM brought their expertise in the orthology area, in order to design the orthology relationship. OG supervised this work. All authors participated in the writing of the manuscript, read and approved the final manuscript.

## Figures and Tables

**Table 1:** Identitag as a tag identifier. This table provides examples illustrating various situations regarding tag identification using Identitag.

			Tag sequence	Transcript identification number	Protein identification number	Protein description
1. Unidentified			CATGACAGCACGGG			

	one EST cluster		CATGAAAATTCTAA	BBSRC603768552F1.1.3.1		
	
2. EST cluster(s)		redundant EST clusters	CATGAGAATAATCT	BBSRC602906088F1.1.3.4 BBSRC602906088F1.1.3.5 AJ508717		
	more than one EST clusters					
		non redundant EST clusters	CATGAAAGACTTCT	12595754.1.3.1 BBSRC603772421F1.1.3.1 AB038230.1.3.477		

3. Annotated (one annotated transcript sequence)			CATGAAAGCCAAGA	J02828	P09207	Tubulin beta-6 chain

4. Annotated (different annotated transcript sequences with the same annotation)			CATGAACTAAAACC	BBSRC603002454F1.1.3.1 BSRC603002454F1.1.3.10	P18937 P18937	NADH-ubiquinone oxidoreductase chain 2 NADH-ubiquinone oxidoreductase chain 2

5. More than one different annotations	redundant annotations		CATGCACTTTGTAT	BBSRC603008189F1.1.3.1 BBSRC603122686F1.1.3.2 AF251344	Q8QG60 Q9NP50 Q8QG52	Cryptochrome 2 Hypothetical protein Cryptochrome 2 (Fragment)
	
	non redundant : multiple match		CATGGTGGTGTGGT	AB038230.1.3.135 gcag0018c.a.01_5.1.3.1	P16039 Q8R4X1	Nucleophosmin Alkaline ceramidase
